# Estabilidad de color de dientes acrílicos inmersos en una solución pigmentante

**DOI:** 10.21142/2523-2754-0904-2021-082

**Published:** 2021-12-09

**Authors:** Jessica Paola Rojas Saldívar, Juan Antonio Díaz Suyo

**Affiliations:** 1 Carrera de Estomatología, Facultad de Ciencias de la Salud de la Universidad Científica del Sur. Lima, Perú. paolarojas955@gmail.com, antonino0410@gmail.com Universidad Científica del Sur Carrera de Estomatología Facultad de Ciencias de la Salud Universidad Científica del Sur Lima Peru paolarojas955@gmail.com antonino0410@gmail.com

**Keywords:** color, diente artificial, café, pigmentación, color, tooth, artificial, coffee, pigmentation

## Abstract

**Objetivo:**

El objetivo de este estudio fue comparar la estabilidad del color de dientes acrílicos inmersos en una solución pigmentante.

**Métodos:**

Se realizó un estudio experimental *in vitro* en 40 dientes acrílicos de dos casas comerciales sumergidas en café y agua destilada (control), divididas en 4 grupos de 10 dientes cada uno, G1 (dientes Duratone-n® sumergidos en agua destilada), G2 (dientes Duratone-n® sumergidos en café), G3 (Dientes Ivostar® sumergidos en agua destilada) y G4 (Dientes Ivostar® sumergidos en café). Los especímenes fueron sumergidos en su respectiva solución durante 10 minutos al día por un periodo de 14 días. Finalmente, las muestras fueron almacenadas en una incubadora a una temperatura constante de 37 °C. El color se registró en dos tiempos (tiempo inicial y a los 14 días) utilizando el espectrofotómetro Vita Easyshade® V. Una vez obtenidos los resultados, se analizaron con el programa Stata 14.

**Resultados:**

Los dientes Ivostar® sumergidos en café (2,24) y agua destilada (2,88) exhibieron los mayores valores de ∆E en comparación con los dientes Duratone-n® sumergidos en café (1,53) y agua destilada (1,60).

**Conclusión:**

Se concluyó que, después de la exposición al café, los dientes de la marca Duratone-n® presentaron mayor estabilidad de color en comparación con los dientes de la marcar Ivostar®.

## Abstract (en)

**Objective:**

The objective of this study was to compare the color stability of acrylic teeth immersed in a pigmenting solution.

**Methods:**

An experimental *in vitro* study was carried out in 40 acrylic teeth from two commercial companies which were immersed in coffee and distilled water (control). The teeth were divided into 4 groups of 10 teeth each: Group-1 (Duratone-n® teeth immersed in distilled water), Group-2 (Duratone- n® immersed in coffee), Group-3 (Ivostar® teeth immersed in distilled water) and Group-4 (Ivostar® teeth immersed in coffee). The specimens were immersed in the respective solutions for 10 minutes a day for a period of 14 days. Finally, the samples were stored in an incubator at a constant temperature of 37 ° C. The color was recorded at two time-points (baseline time and at 14 days) using the Vita Easyshade® V spectrophotometer. The results were analyzed using the Stata 14 program.

**Results:**

Ivostar® teeth immersed in coffee (2.24) and distilled water (2.88) showest the highest ∆E values compared to Duratone-n® teeth immersed in coffee (1.53) and distilled water (1.60).

**Conclusion:**

After exposure to coffee, the Duratone-n® brand teeth presented greater color stability compared to those of the Ivostar® brand.

## INTRODUCCIÓN

El uso de prótesis parciales y totales son alternativas de tratamiento integral en rehabilitación oral ^1^^,^^2^ nos permite devolver la estética, la autoestima y la capacidad para socializar entre los pacientes que hacen uso de ellas ^3^^,^^4^. Los dientes artificiales de resina acrílica que se usan en estas prótesis deben presentar múltiples propiedades beneficiosas como resistencia, deformabilidad, eficacia masticatoria, estética, estabilidad del color, dificultar la adsorción, dificultar la formación de placa bacteriana, no producir olores, biocompatibilidad, fácil manipulación y mejor adherencia a la base de la prótesis ^5^.

En el mercado encontramos distintas marcas de dientes de acrílico, como Duratone-n® e Ivostar®, que son las marcas que utilizaremos en el presente estudio, con distintos números de capas de resina acrílica. Duratone-n® son dientes de resina acrílica con cuatro capas, dos capas de gingival que les dan el color, van en el interior de diente y simulan la dentina, y dos capas de incisal, que es la capa exterior que simula el esmalte dental y les da translucidez ^6^. Por otro lado, los dientes Ivostar® tienen tres capas de resina acrílica, lo cual les confiere la armonía de los dientes originales ^7^.

Un defecto importante en este tipo de dientes es la inestabilidad de color, que puede deberse a factores externos. ^3^^,^^8^^-^^10^. El color de los dientes acrílicos se puede alterar comúnmente, pues a diario los pacientes portadores de prótesis consumen diferentes alimentos y bebidas ^11^^,^^12^, entre ellos el café, que es una de las bebidas más consumidas en el mundo y que posee pigmentos que pueden alterar la estabilidad de color, como los carotenoides que causan su color peculiar. Estudios previos han demostrado que algunas bebidas como el té, el café, los jugos de fruta y el vino pueden causar coloración en los materiales dentales ^2^^,^^5^^,^^8^^,^^9^^,^^13^. 

Mousavi ^8^ evaluó la estabilidad de color de varios tipos de dientes acrílicos expuestos a café, té y cola, y observó que el café mostró un mayor cambio de color en los dientes acrílicos Apple (Ideal Macou, Tehrán, Irán). En su tesis, Quinapaxi ^14^ evaluó la variación del color debido a las soluciones pigmentantes (soda naranja, té y café) en dientes de acrílico, y obtuvo como resultado que la sustancia que produjo mayor cambio de color en los dientes de acrílico fue el café.

La etiología de la decoloración de los dientes acrílicos es multifactorial, puede deberse al desgaste, la falta de una correcta higiene por parte del paciente, la exposición a manchas y el tiempo ^15^. Estos cambios en el color se pueden medir mediante métodos clínicos o técnicas instrumentales como es la espectrofotometría. El uso del espectrofotómetro es más preciso y reduce errores en comparación con la medición realizada por el ojo clínico ^3^.

Existen pocos estudios relacionados con las distintas marcas de dientes artificiales y su estabilidad de color; por tanto, el objetivo de este estudio es comparar la estabilidad del color entre los dientes acrílicos de las marcas Duratone-n® e Ivostar® sumergidos en café.

## METODOLOGÍA

Este estudio fue aprobado por el Comité Institucional de Ética en Investigación de la Universidad Científica del Sur (CIEI-Científica) con el código N.º 493-2019-PRE8.

El estudio fue de tipo experimental *in vitro*. Para determinar el tamaño de la muestra, se utilizó la fórmula de comparación de dos medias a un nivel de confianza del 95%, con poder estadístico al 80%, precisión de 1,44 y varianza obtenida en la prueba piloto de 1,27, lo que da como resultado 8 dientes por grupo, pero se decidió redondearlo a 10 dientes por grupo. Para este estudio, se emplearon 40 incisivos centrales superiores de los dientes de acrílico de la marca Duratone-n® e Ivostar® divididos en 4 grupos de 10 dientes cada uno según la marca y la sustancia a la que se expuso. El primer grupo tuvo 10 incisivos centrales superiores de la marca Duratone-n® sumergidos en agua destilada (control), el segundo grupo tuvo 10 incisivos centrales superiores de la marca Duratone-n® sumergidos en café, el tercer grupo tuvo 10 incisivos centrales superiores de la marca Ivostar® sumergidos en agua destilada (control) y el cuarto grupo tuvo 10 incisivos centrales superiores de la marca Ivostar® sumergidos en café.

Fueron incluidos en el estudio incisivos centrales superiores de los dientes de acrílico de la marca Duratone-n®, color 2A, forma N12, e incisivos centrales superiores de los dientes de acrílico de la marca Ivostar®, color 2A, forma 42. Fueron excluidos los dientes con burbujas, rayados o fisurados.

• Estabilidad de color. Diferencia de color en la superficie de los dientes acrílicos obtenido antes y después de la exposición al café, medida a través del uso del espectrofotómetro Vita Easyshade V.
• **Dientes acrílicos.** Dientes de acrílico (incisivos centrales superiores) color 2A, de forma cuadrangular, utilizados en el presente ensayo experimental, cuyo indicador fueron las marcas comerciales Duratone-n® e Ivostar®.
• **Sustancia de inmersión**. Sustancias en las que se sumergió los dientes de acrílico anterosuperiores, que fueron agua destilada, para el grupo control, y café.
• **Tiemp**o. Periodo en el cual se realizó el estudio y se evaluó el color de los dientes de acrílico en un tiempo inicial y a los 14 días después de sumergirlos en sus respectivas sustancias.

Se utilizaron 40 dientes artificiales de resina acrílica del sector anterior superior (incisivos centrales superiores) de las marcas comerciales Duratone-n® (color 2A, forma N12) e Ivostar® (color 2A, forma 42), según los criterios de selección antes mencionados.

Se procedió a retirar los incisivos centrales superiores de su respectiva tableta; posteriormente, se retiró el excedente de cera de cada diente con la ayuda de una espátula de cera.

Se distribuyeron 10 dientes de acrílico por cada grupo en cuatro envases de vidrio color ámbar. A cada uno se les asignó una identificación: G1 para el grupo control (dientes de la marca Duratone-n® sumergidos en agua destilada), G2 (dientes acrílicos de la marca Duratone-n® sumergidos en café), G3 para el grupo control (dientes acrílicos de la marca Ivostar® sumergidos en agua destilada) y G4 (dientes acrílicos de la marca Ivostar® sumergidos en café).

La sustancia pigmentante se preparó de la siguiente manera: café instantáneo (Nescafé® Tradición Perú) preparado de acuerdo con las instrucciones del fabricante (2 g de café en 200 ml de agua destilada). En un vaso de precipitado de 400 ml, se procedió a mezclar el agua destilada junto con el café y se agitó con una varilla de vidrio.

Los especímenes fueron sumergidos en su respectiva solución durante 10 minutos al día por un periodo de 14 días ^(16, 17)^. Finalmente, las muestras fueron almacenadas en agua destilada en una incubadora en el laboratorio de microbiología de la Universidad Científica del Sur, a una temperatura constante de 37 °C. Antes de realizar la medición del color, las muestras se lavaron con agua destilada y fueron secadas con papel absorbente. 

El color se registró en dos tiempos (tiempo inicial y a los 14 días) utilizando el espectrofotómetro Vita Easyshade® V. Cada diente se manipuló utilizando guantes y una pinza para algodón para evitar la contaminación del cuerpo de prueba. Al comenzar con el registro del color, se verificó que Vita Easyshade® estuviera debidamente cargado y se procedió a la calibración automática del aparato. Una vez realizada la calibración, apareció el menú de medición y el aparato estuvo listo para su uso. La calibración se realizó cada 10 muestras y se seleccionó el símbolo de medición de color básico. La medición se realizó por un investigador previamente capacitado y calibrado. Las muestras se colocaron sobre una superficie blanca en un ambiente con luz natural. La punta lectora del espectrofotómetro digital se colocó directamente sobre la cara vestibular de la muestra, en el tercio medio de cada diente. Se pulsó el botón de medición hasta que sonaron tres tonos seguidos, que indicaron la finalización del proceso de medición.

Se tomó dos registros de color por muestra y el valor de esa muestra fue el promedio de las dos mediciones.

La diferencia del color (∆E) entre un color inicial y la subsecuente medición se calculó empleando la siguiente fórmula:

∆E = [(L*1 - L*0)2 + (a*1 - a*0)2 + (b*1 - b*0)2]1/2

Los datos se registraron en una tabla de recolección de datos. Una vez obtenidos los resultados, se realizó la tabulación de los datos recolectados en el programa Microsoft Excel 2013 para luego ser analizados en el programa Stata 14. La normalidad de los datos numéricos (estabilidad de color) se evaluó mediante la prueba de Shapiro-Wilk. Se realizó un análisis descriptivo de la variable numérica (estabilidad del color) mediante los cálculos de la media, desviación estándar, varianza, valor mínimo y valor máximo. Para comparar la estabilidad de color de los dientes acrílicos inmersos en café y de cada marca en los 2 tiempos de evaluación, se usó la prueba t de Student para muestras independientes. El nivel de significancia se estableció al 5%.

## RESULTADOS

En la Tabla 1 se observa el análisis descriptivo de los valores de ∆E para las dos marcas de dientes sumergidos en 2 sustancias. Se encontró que los dientes de la marca Duratone-n® presentan menor cambio de color en comparación con los dientes de la marca Ivostar® al ser sumergidos en café y agua destilada después de 14 días.

**Tabla 1:** Análisis descriptivo de los valores de ΔE entre los dientes acrílicos de la marca Duratone-n® e Ivostar®, sumergidos en café y agua destilada.

Marca	Sustancia	N.°	Media	Desviación estándar	Mediana	Varianza	Valor mínimo	Valor máximo
Duratone-n^®^	Café	10	1,53	0,39	1,38	0,15	1,03	2,29
	Agua destilada	10	1,60	1,06	1,36	1,13	0,56	4,21
Ivostar^®^	Café	10	2,24	1,27	2,67	1,61	0,51	3,91
	Agua destilada	10	2,88	1,08	2,66	1,17	1,18	5,04

Los resultados de la prueba t de Student para muestras independientes con varianzas desiguales no mostraron diferencia significativa entre los valores ∆E de los dientes artificiales Duratone e Ivostar después de 14 días de inmersión en café, donde P = 0,121 (Tabla 2).

**Tabla 2:** Media y desviación estándar de los valores ΔE de los dientes de acrílico de la marca Duratone e Ivostar sumergidos en café y agua destilada después de 14 días

Sustancia	Marca de dientes	Media± DE	P
Café	Duratone-n^®^	1,53 ± 0,39	0,121*
	Ivostar^®^	2,24 ± 1,27	
Agua destilada	Duratone-n^®^	1,60 ± 1,06	0,01**
	Ivostar^®^	2,88 ± 1,08	

*Prueba t de Student para muestras independientes con varianzas desiguales

** Prueba U de Mann-Whitney

Los resultados de la prueba U de Mann-Whitney mostró diferencia significativa entre los valores ∆E de los dientes artificiales Duratone e Ivostar después de 14 días de inmersión en agua destilada, donde P = 0,01 (Tabla 2).

## DISCUSIÓN

La estabilidad de color de los dientes de acrílico es un elemento importante en la estética de una prótesis dental. Ya que están en constante exposición a alimentos y bebidas que pueden pigmentarlos, es necesario que estos materiales presenten una buena estabilidad de color a largo plazo. El objetivo de este estudio fue evaluar la estabilidad de color de dos marcas de dientes de acrílico después de ser sumergidos en una sustancia pigmentante, con la ayuda de un espectrofotómetro.

Dentro de la composición química de los dientes acrílicos de marca Duratone-n® tenemos polimetacrilato de metilo, etilenglicol dimetacrilato, fluorescencia y pigmentos ^6^, en comparación con la marca Ivostar® que, en su hoja de datos, solo refiere como composición química dientes de polímero síntetico hecho con polimetilmetacrilato ^7^. Allí radica una clara diferencia entre la composición química de cada marca de dientes y la diferencia encontrada en los resultados se debió a esto. El uso de materiales hidrófobos para la fabricación de los dientes de resina acrílica es un factor importante en el cambio de color de estos dientes, ya que puede aumentar la estabilidad del color ^11^.

Estudios previos señalan que los valores de ∆E ≤ 3,3 son clínicamente aceptables ^18^^,^^19^. De acuerdo con el presente estudio, 14 días después de la inmersión en las respectivas sustancias, las dos marcas de dientes de acrílico mostraron cambios de color clínicamente aceptables (∆E ≤ 2,8). Mousavi *et al*. ^8^ realizaron un estudio *in vitro* en el que evaluaron y compararon los cambios de color de tres marcas de dientes de acrílico (Apple, Irán; Ivoclar, Italia; y PolyDent, Eslovenia), sumergidos en té, bebida carbonatada (cola) y café, durante 1,3 y 6 semanas. El estudio mostró resultados similares a la presente investigación en la primera semana, en que los dientes de acrílico fueron sumergidos en café con valores de ∆E ≤ 2,7. Roslan *et al*. ^20^ realizaron un estudio en el que evaluaron los cambios de color de una marca de dientes de acrílico sumergidos en cúrcuma y café. Los cambios de color se midieron en intervalos de 1, 7 y 15 días. El estudio mostró un ligero cambio de color después de los días 7 y 15 para los dientes sumergidos en café, en comparación con los dientes sumergidos en cúrcuma, en el cual los cambios en el color fueron bastante visibles. Al igual que en este estudio, los dientes sumergidos en café presentan un ligero cambio en el color que es clínicamente aceptable. 

Ansari *et al*. ^21^ compararon la estabilidad de color de 2 marcas de dientes artificiales (Glamour e Ideal Dent) sumergidos en té y café. Los resultados mostraron que los de la marca Glamour presentaron mayor cambio de color en comparación con la marca Ideal Dent; sin embargo, ambos cambios de color no excedieron los valores ∆E mayores a 3,3, por lo cual resultaron clínicamente aceptables. En nuestro estudio, del mismo modo, los dientes de la marca Ivostar presentaron un cambio mayor en comparación con los de la marca Duratone, pero ninguno excedió los valores ∆E clínicamente aceptables. 

Gregorius *et al*. ^15^ evaluaron la estabilidad de color de tres marcas diferentes de dientes acrílicos sumergidos en agua destilada, café y vino tinto. Los resultados mostraron que, después de 7 días, los cambios en el color en el caso del agua destilada son ∆E ≤1,3 y en el del café, ∆E ≤2.9, como muestran los demás estudios comparados. En su mayoría, podemos observar que los valores ∆E no exceden los valores clínicamente aceptables. Hipólito *et al*. ^11^ evaluaron el efecto de diferentes bebidas sobre la degradación del color de diez marcas de dientes de acrílico sumergidas en saliva, café, bebida carbonatada y jugo de naranja, en tiempos inicial, 7, 15 y 30 días, y los dientes acrílicos mostraron diferentes cambios de color por cada solución. La solución del café mostró cambios de color en un intervalo de 15 días (0,98-2,20), en comparación con el presente estudio, en un intervalo de 14 días (1,53-2,24). Sin embargo, ambos estudios mostraron cambios de color clínicamente aceptables.

Una de las principales limitaciones del presente estudio fue que, para el grupo control, se utilizó agua destilada. Al inicio se quiso emplear saliva artificial para asemejar lo más posible lo que sucede en boca, pero al desarrollarse durante la pandemia de la COVID-19 fue difícil la disponibilidad de esta durante ese tiempo.

Las soluciones se almacenaron a 37 °C, lo que simula la temperatura de la cavidad oral; sin embargo, sabemos que el café se ingiere caliente en la mayoría de los casos, lo que puede influir en el cambio de color. Se sugiere tomar en cuenta estas limitaciones para futuros estudios y se invita a realizar estudios longitudinales que evalúen el cambio de color de los dientes artificiales de las prótesis en pacientes en un periodo prolongado para obtener información más certera y evaluar el comportamiento de estos materiales *in vivo.*

## CONCLUSIONES

Considerando el ∆E ≤ 3,3 como el cambio de color clínicamente aceptable, parece que, a pesar del cambio de color en ambos grupos de dientes, están dentro del rango clínicamente aceptable. Después de la exposición al café, los dientes de la marca Duratone-n® presentaron igual estabilidad de color en comparación con los dientes de la marcar Ivostar®.

## Fuente de financiamiento:

Autofinanciado
